# Real-world performance of large-scale propensity score adjustment strategies: Matching, weighting, and stratification

**DOI:** 10.21203/rs.3.rs-9987133/v1

**Published:** 2026-07-01

**Authors:** Kelly M Li, Martijn J Schuemie, Patrick B Ryan, Linying Zhang, Yong Chen, Kashish Priyam, Nicole Pratt, George Hripcsak, Marc A Suchard

**Affiliations:** 1 Department of Biostatistics, University of California, Los Angeles, Los Angeles, CA, USA; 2 Janssen Research and Development, Raritan, NJ, USA; 3 Department of Biomedical Informatics, Columbia University Medical Center, New York, NY, USA; 4 Institute for Informatics, Data Science and Biostatistics, Department of Medicine, Washington University in St Louis, St Louis, Missouri, USA; 5 Department of Biostatistics, Epidemiology and Informatics, Perelman School of Medicine, University of Pennsylvania, Philadelphia, PA, USA; 6 Quality Use of Medicines and Pharmacy Research Centre, University of South Australia, Adelaide, Australia; 7 VA Informatics and Computing Infrastructure, US Department of Veterans Affairs, Salt Lake City, UT, USA; 8 Department of Human Genetics, University of California, Los Angeles, Los Angeles, CA, USA

**Keywords:** propensity score, real-world data, observational study

## Abstract

**Objective:**

Propensity score (PS) models are commonly used for addressing confounding in observational studies. Researchers are increasingly incorporating large numbers of covariates into PS models, using techniques like large-scale propensity score (LSPS) adjustment. It remains unclear if the inclusion of many covariates affects the subsequent choice of adjustment strategy.

**Methods:**

In this paper, we evaluate commonly used adjustment strategies using large-scale models, including matching, stratification, and inverse probability of treatment weighting (IPTW), to estimate treatment effects under a new-user cohort design. Evaluations employ real-world data across four national healthcare databases and incorporate 3,840 treatment effect estimates per model specification for 24 different treatment cohort comparisons against 160 negative control outcomes. We assess adjustment strategies for covariate balance between treatment cohorts and type-I and type-II error, mean-squared error, and precision of the adjusted effect estimate.

**Results:**

Across all model specifications, IPTW with symmetric Crump trimming and 1:1 matching perform best overall, achieving strong balance and low bias. Additionally, empirical calibration of effect estimates can substantially reduce differences between strategies.

**Discussion:**

No single strategy is clearly superior in all settings. We recommend incorporating the top-performing strategies at least as sensitivity analyses in studies even if researchers anticipate that some other strategy may excel due to expectation of their study’s operating characteristics. We also recommend use of diagnostics like covariate balance to check strategy performance.

**Conclusion:**

IPTW with Crump trimming and 1:1 matching are strong default choices for large-scale PS adjustment, but strategy selection should be guided by study context and validated with balance and error diagnostics. We broadly recommend empirical calibration to reduce sensitivity to model choice.

## 2 Introduction

### 2.1 Observational studies and propensity scores

Observational healthcare data are increasingly being utilized to estimate and evaluate treatment effect models in real-world populations. In recent years, the growing availability of administrative claims and electronic health record (EHR) data has enabled large-scale studies that span millions of patients with tens of thousands of covariates and long follow-up periods [1, 2]. Using large scale data provides a wealth of opportunities to provide evidence about treatment effects that can meaningfully inform healthcare decisions [3], but researchers are often faced with methodological challenges, particularly in controlling for confounding.

To study the comparative effectiveness or safety of alternative treatments, the new-user cohort method attempts to emulate randomized clinical trials using observational data [4]. At the core, the design compares subjects who initiate a treatment of interest (the target cohort) against those who initiate a control treatment (the comparator cohort). Typically, outcomes are measured during a pre-defined follow-up length of time to estimate the relative effect of the target treatment compared with the comparator.

In the absence of randomization, patients in one treatment group may systematically differ from those in another treatment group, potentially biasing treatment effect estimates. Propensity score (PS) methods are a technique commonly used to address this issue [5, 6]. The PS is defined as the baseline probability that a given patient will receive the target treatment over the comparator, and scores are commonly estimated using binary regression models. Once estimated, patients in both treatment groups can be matched, stratified, or weighted by their PS in order to control for confounding and make the groups more comparable.

Typically, PS models contain a small (between, e.g., 10–50), hand-picked set of covariates that researchers believe are related to the outcome. Recent advances in observational research by the OHDSI [7] community have made it feasible to estimate PS models using extremely large sets of patient covariates. These large-scale PS (LSPS) models typically include millions of patients and tens of thousands of covariates that summarize demographic information, pre-existing conditions, drug exposures, measurements, and medical procedures. These patient characteristics are not selected based on any prior information, and are rather designed to be highly generic. Due to the large dimensionality of this method, large-scale PS models are typically fit using large-scale regularized regression [8]. While this approach has been previously found to improve PS model fit and balance treatment groups [9, 3, 10], there is a notable lack of empirical evaluation of how different PS adjustment strategies (matching, stratification, weighting) perform in real-world scenarios.

### 2.2 Matching and stratification

Matching and stratification are two of the most widely used strategies for adjusting for confounding once the PS is fit. Matching attempts to construct comparable treatment groups by matching patients in the target cohort to those in the comparator cohort who have similar propensity scores. Typically, a recommended caliper of 0.2 standard deviations of the logit-transformed propensity score [11] is used to ensure high-quality matches, where patients are only matched if their PS fall within the specified caliper. Matching is also defined by the ratio of people who get matched between cohorts. In fixed-ratio matching, each treated individual is matched to a predetermined number of comparator subjects (e.g., one-to-one or one-to-many). In contrast, variable-ratio (VR) matching allows the number of comparator matches per treated individual to vary depending on the availability of suitable matches. Increasing the number of matches per treated subject can improve precision as each matched group increases in sample size. However, as additional matches are included, the similarity between matched subjects may decrease, which can introducing additional bias. Rassen et al. [12] found that, in simulations involving 50,000 patients and eight covariates, variable-ratio matching achieved the highest precision with relatively small increases in bias when compared to to fixed-ratio approaches.

Stratification is another commonly used method for incorporating PS into a treatment effect estimation. Instead of matching individuals directly, patients are grouped into strata according to their estimated PS values. Typically, it is recommended to use five strata when stratifying by PS [13]. A limitation of this approach is that individuals within the same stratum are treated as having the same propensity score, which can lead to residual bias within strata.

In 1:1 matching, each treated subject has exactly one match, so the pairwise comparison inherently accounts for the matching in the eventual outcome model. When matching at ratios beyond 1:1 (such as variable ratio matching), or when using stratification, the variance of the effect estimate needs to properly reflect the clustered structure of the data. To do so, we condition the outcome model on the matched sets or strata rather than including the set or stratum indicators as covariates. These models are reported as ‘Conditional’ in Table 3, emphasizing that they explicitly respect the design structure required by matching or stratification.

### 2.3 Inverse propensity score treatment weighting

Another widely used PS adjustment approach is inverse probability of treatment weighting (IPTW). In IPTW, each patient is assigned a weight equal to the inverse of the probability of receiving the treatment they actually received [14, 15]. A common challenge with IPTW is that it struggles under extreme weights. Patients who are very unlikely to receive the target and ended up receiving the target anyway will have very heavy inverse weights, as will patients who were very likely to receive the target but ended up receiving the comparator. There are multiple ways to handle extreme weights: researchers can opt to trim away the problematic patients based on their raw PS, left or right censor extreme weights, or use overlap weighting schemes [16, 17]. In this study, we compare two ways of trimming by PS, and overlap weighting.

All trimming methods can be parametrized using the trimming fraction (10% in this study). The first approach is proposed by Crump et al. [18], which we dub IPTW (Crump), where observations in the upper and lower trim fractions of the PS distribution are removed, agnostic of treatment assignment. The second approach uses a two-step method proposed by Stürmer et al. [19], which we now call IPTW (Stürmer). In the first step, observations in the non-overlapping PS zones between groups are removed, thereby removing all patients who are not likely to have a comparable counterpart in the other treatment group. Then, we remove the trim fraction of patients whose propensity scores are most contrary to the treatment they actually received. Overlap weighting [16, 17] up-weights patients in the region of greatest PS overlap between groups, naturally avoiding extreme weights without explicit trimming.

Each strategy targets a different causal estimand: matching targets the average treatment effect on the treated (ATT), stratification targets the average treatment effect (ATE), overlap weighting targets the average treatment effect on the overlap population (ATO), and IPTW with trimming targets the ATE within the trimmed population [20, 21, 17].

### 2.4 Existing benchmarks

There exists previous work that compares some commonly-used PS adjustment strategies in a large-scale context [22], which found that IPTW with trimming was greatly underperforming in comparison to other techniques like matching and stratification. Recommendations were made for 1:1 and VR matching, but the overall conclusions suggested that the choice of optimal PS model is highly nuanced and dependent on its use case. Small-scale simulation studies have also previously favored IPTW for bias control and precision, though these findings may not generalize to the large-scale settings [23, 24, 25]

Despite the adoption of LSPS models in large-scale observational health studies [1, 2], there remains limited empirical evidence that directly compares a large set of adjustment strategies for PS models across many options for matching ratios, strata counts, and trimming methods for IPTW. To address this, we developed the Survey of Propensity-score Adjustment Routes for Treatment-effect Analyses (SPARTA) initiative. SPARTA is a large-scale, systematic evaluation of PS model specifications using real-world data with over 11 million patients across four observational health databases. We hope that the insights gained from this work will help guide decision-making for PS model design in observational health studies.

## 3 Methods

### 3.1 Study design and data sources

We conducted a comparative new-user cohort study to evaluate LSPS adjustment strategies in real-world observational data. We selected a representative case study by targeting new users of first-line monotherapies treating hypertension, with a focus on individual drugs and their corresponding classes, in accordance with the 2017 AHA/ACC guidelines [26]. Patients were defined as new users if they received their first observed hypertension treatment and had a documented hypertension diagnosis at or within one year prior to treatment initiation [1]. Detailed definitions for the cohorts defining the treatments of interest can be found in Supplement A.

Of the many first-line monotherapies, we selected four classes of interest for class-level comparisons and one representative drug per class for drug-level comparisons. In order to meaningfully evaluate the different PS adjustment strategies, target/comparator choices were selected to provide sufficient baseline confounding to meaningfully evaluate PS adjustment (details in Supplement B).

This study uses four federated data sources spanning 2000–2026, identifying a total of 11,433,652 patients with hypertension who initiated the treatments of interest. Across databases, covariates that were very rare, defined as appearing in less than .1% of the subjects, are removed, leading to a non-constant covariate count across databases. In general, each model hovered around 12, 000 defined covariates per PS model.

Table 1 describes the federated data sources in this study, and Table 2 describes the patient counts for those meeting the criteria for hypertension and treated with each treatment class or drug of interest.

### 3.2 Empirical evaluation framework

In this study, we utilize an empirical evaluation framework that quantifies bias and variance directly using real-world data. This approach hinges on the usage of 160 negative control outcomes, which serve as a gold standard for evaluating PS performance [1, 22]. Negative controls are defined as outcomes where exposure to either the target or comparator treatment is not expected to influence the outcome, such that the null hypothesis is that the effect size is zero. Given our predefined treatment pairs (Section 3.1), we used existing negative control outcomes from existing literature [1], selected using a combination of automated algorithms and manual review [27].

To complement the negative controls and evaluate non-null effects, we generated a set of three synthetic positive controls with effect sizes [1.5, 2, 4] for each negative control. Positive controls were generated by first fitting the model for estimated effect size on the negative control, and then shifting the likelihood of the estimate by the targeted positive effect size.

### 3.3 Propensity score adjustment schemes

We applied a broad set of PS adjustment strategies to benchmark using large-scale real-world data. We first fit the PS model, and then fit Cox proportional hazards model for a the risk of each negative outcome of interest, conditioned on the PS. Each method is evaluated across 12 treatment comparisons and 160 outcomes, resulting in 1,920 outcome models per database. Table 3 summarizes all PS adjustment techniques evaluated in this study and whether the outcome models were conditioned on matched sets or strata.

Covariates for the PS models were selected using a comprehensive and systematic approach, intended to create as broad a representation of patient baseline characteristics as possible, irrespective of whether or not they were known confounders. The models included 10^4^–10^5^ covariates covering pre-treatment patient demographics, medical history, prior drug exposures, and healthcare utilization patterns [9].

To remove obvious instrumental variables, otherwise known as covariates that are highly correlated with the treatment while being unlikely to be related to the outcome, we numerically compute univariate correlation between each covariate and the treatment. If the highly correlated covariate is not causally related to the outcome based on existing domain knowledge, the variable is removed. Detailed covariate classes and selection procedures for LSPS models are provided in Supplement D.

We note that we investigate matching with a fixed ratio at 1:25, and also stratification into 25 strata. These are unusual group sizes typically not seen in traditional PS-adjusted studies, and we include them in this study as our data sources are distinctly large in scale. This allowed us to examine how expanding matches or strata to larger-than-normal sizes can influence the performance of PS adjustment strategies.

All matching in this study used recommended calipers of 0.2 standard deviations on the logit of the PS [28]. Extreme weights in IPTW were mitigated using the previously described trimming methods (Crump, Stürmer) with a trim ratio of 10%, or using overlap weighting [29, 16].

All propensity scores were estimated using large-scale *L*_1_-regularized logistic regression with hyperparameters selected via 10-fold cross-validation [8]. The analyses in this study were implemented using R packages from the Health Analytics Data-to-Evidence Suite (HADES) [30], ensuring reproducibility and transparency. The full codebase is open-source and publicly available at GitHub.

### 3.4 Metrics

As previously discussed in Section 3.2, we use an empirical evaluation framework to assess the performance of LSPS models using negative and positive control outcomes [31, 22]. Each analysis is defined as one target/comparator/outcome tuple, and produces an estimated treatment effect for the outcome of interest, accompanied by a 95% confidence interval and a two-sided p-value testing the null hypothesis of no effect.

We apply an empirical calibration procedure developed by Schuemie et al. [31] to all of our estimates, which adjusts p-values and confidence intervals to near-nominal values using the observed distribution of effects for the control outcomes [32]. Empirical calibration constructs a null distribution using all negative control estimates, with the goal of capturing both random and systematic error. To prevent overly optimistic results, this is done using a leave-one-out approach, such that the empirical null is estimated excluding the control currently under evaluation. P-values are then calibrated using the empirical null, rather than the typically used asymptotic, theoretical null (typically a Gaussian centered on 0 on the log-scale of relative risk / hazard).

Overall, using our negative and positive control framework, we calculate the following metrics for each PS adjustment strategy across target/comparator pairs, both before and after empirical calibration:

- Type-I error: The proportion of negative controls for which the null hypothesis was incorrectly rejected (*α* = 0.05), equivalent to the false positive rate.
- Type-II error: The proportion of positive controls for which the null hypothesis was not rejected (*α* = 0.05), representing the false negative rate.
- Mean precision: The geometric mean of precision across negative controls, calculated as $\frac{1}{SE^{2}}$, where *SE* is the estimated standard error of the treatment effect.
- Mean squared error (MSE): The mean squared difference between estimated and true effect sizes across all control outcomes.
- Maximum absolute standardized mean difference (SMD): After PS adjustment, the maximum absolute standardized difference in covariate means among the covariates adjusted for in the PS models between target and comparator groups. Covariates with an absolute difference above 0.1 are flagged as imbalanced.

Among these listed metrics, we focus on SMD, which serves as a primary metric for assessing the ability of the PS adjustment to make treatment groups comparable [3]. We also emphasize Type-I error for evaluating bias, such that we have an overall evaluation approach to provide a data-driven method for quantifying the accuracy of LSPS models in real-world health data.

## 4 Results

SPARTA integrates longitudinal claims and electronic health records data from four independent data sources, encompassing over 11 million patients, to provide a large-scale benchmark of different LSPS model specifications. In this study, we evaluate 9 different PS model specifications across 12 distinct treatment pairs for 160 negative control outcomes in each of 4 databases, leading to a total of 9 * 12 * 160 * 4 = 69120 hazard ratio estimates. Our primary evaluation focus is on aggregate metrics across all comparisons, including both class-vs-class and drug-vs-drug analyses, and we select the target cohort to be smaller than the comparator cohort.

### 4.1 Balance

Our first metric of interest is the ability of each PS adjustment strategy to balance covariates between the target and the comparator cohorts such that subjects become comparable to each other. To quantify this, we aggregate balance metrics across target–comparator pairs and report the maximum absolute SMD for each pair across all four data sources.

**Figure 1:**
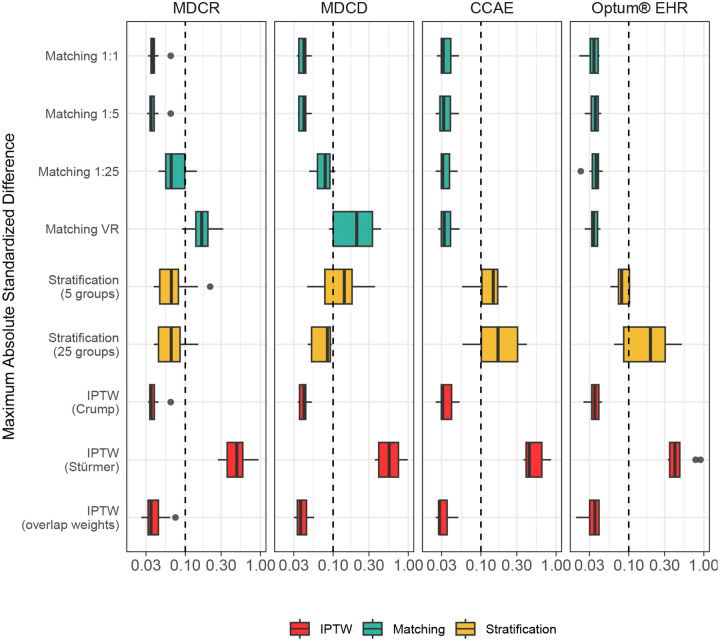
Box plot of maximum absolute standardized mean difference between target and comparator cohorts for covariates adjusted for in PS models across all comparisons. A dashed horizontal line at 0.1 indicates the cutoff for acceptable balance. Values above the dashed line indicate poor balance, while those below the dashed line indicate sufficient balance.

Figure 1 shows the maximum absolute SMDs for covariates included in all PS models of interest, stratified by database. Results indicate that matching with ratios of 1:1 and 1:5, along with IPTW (Crump) and IPTW (overlap weights), showcased the lowest SMD values below the nominally recommended 0.1, showing acceptable balance was achieved. The two largest databases, CCAE and Optum^®^ EHR, also presented low SMD values for matching with variable-ratio and fixed 1:25. Balance was not adequately achieved with IPTW (Stürmer), and was only sometimes achieved with stratification.

Table 4 displays the SDMs for select covariates of interest before and after 1:1 matching and IPTW (Stürmer), illustrating one model with strong balance and one with weak balance. We selected baseline characteristics, including demographic information, and also specific general medical history traits that exhibited poor balance after adjustment. The 1:1 matching model achieves near-complete balance below the 0.1 threshold, while IPTW (Stürmer) struggles more, though both strategies improve comparability relative to baseline.

### 4.2 Type-I error

**Figure 2:**
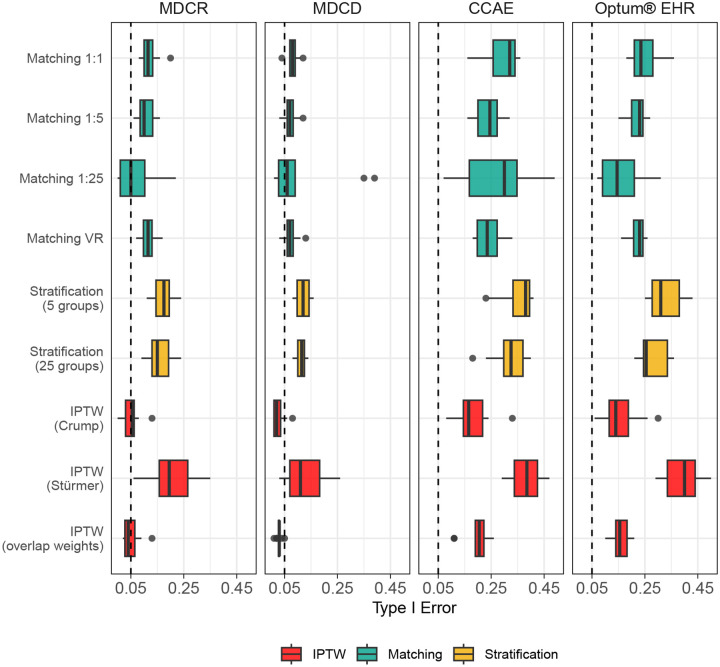
Box plot for type I error without calibration for classwise comparisons across databases for all propensity score models. A vertical dashed line is presented at the nominal type-I error rate of 0.05.

Figure 2 shows the type-I error between PS models across all analyses of interest and all databases. Results indicate that IPTW (Crump) and overlap weighting achieved the lowest type-I error rates, while IPTW (Stürmer) had the highest error rate across all databases. Matching showed mixed results when selecting different ratios to match on. 1:25 outperformed in MDCR and Optum^®^ EHR, but showed higher variance in CCAE where 1:5 and VR matching had lower median error and smaller spread. We also note that MDCD was similar across all matching ratios. Increasing the number of strata for stratified models did not significantly impact error rates across all databases.

### 4.3 Detailed metrics

**Figure 3:**
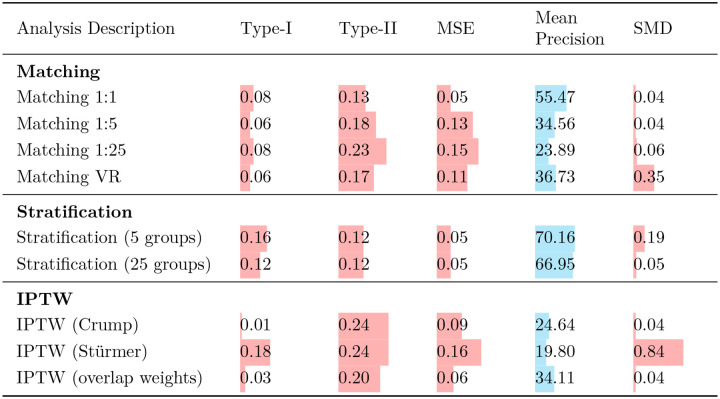
Evaluation metrics of all PS models for new users of metoprolol vs. lisinopril in the Merative Marketscan Medicaide Supplemental Database (MDCD). Columns plotted in red correspond to metrics for which lower values indicate better performance, while columns in blue correspond to metrics for which higher values indicate better performance.

In this study, our main focus was on reporting aggregate metrics of interest, namely balance and type-I error rates. We also computed secondary metrics of interest, including mean precision and type-II error, to assess how the PS models perform in distinguishing between negative and positive control outcomes when estimating treatment effects. Figure 3 shows all evaluation metrics in MDCD for one specified analysis comparing metoprolol (target) vs lisinopril (comparator).

By laying out all the metrics of interest, we are able to closely examine methods that may have previously performed similarly in our primary error metrics, type-I error and SMD. Specifically, we note that while fixed-ratio matching and IPTW with Crump trimming or overlap weights exhibited low type-I error and exceptional balance, 1:1 matching had the highest precision and lowest MSE. Generally, stratification had the highest precision, with the trade-off of having higher type-I error.

### 4.4 Calibration

**Figure 4:**
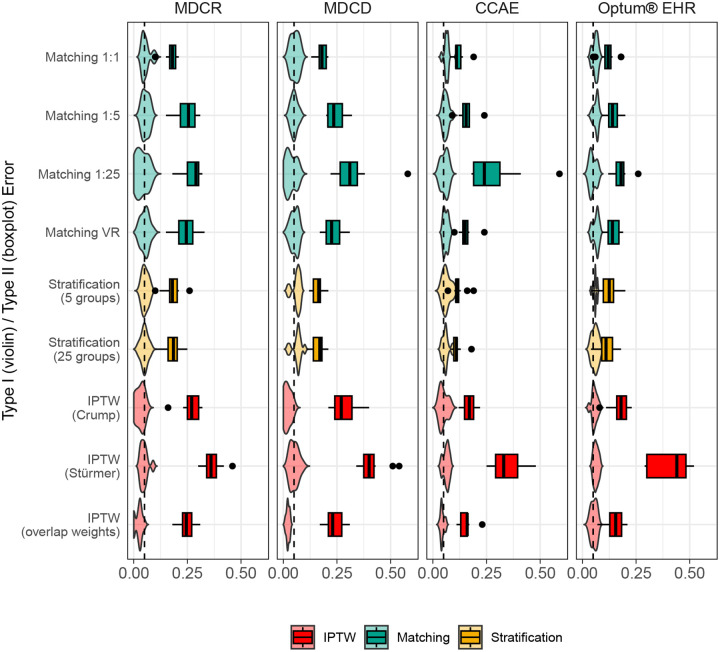
Box plot for type II error overlayed on a violin plot for type I error, post-calibration, across databases for PS models. A horizontal dashed line is presented at the nominal type-I error rate of 0.05. One outlier (value = 0.66, IPTW (Stürmer), Optum^®^ EHR) was excluded from the figure for visualization.

We now present in Figure 4 aggregate type-II errors across all comparisons after calibrating for a nominal type-I error of 0.05. Given that all the type-I error rates are calibrated to the same nominal value, we are able to directly compare type-II errors. While IPTW (Stürmer) still falls out of line and has the highest error rates of all methods, we see a convergence in other models’ performances. In fact, post-calibrated stratification shows a significant reduction in error rates, and ranks among the lowest across the strategies evaluated. IPTW variants show slightly increased type-II error rates in comparison to other strategies, despite being some of the better performers pre-calibration. Generally, calibration tends to converge the performance of the different models toward similar levels.

**Figure 5:**
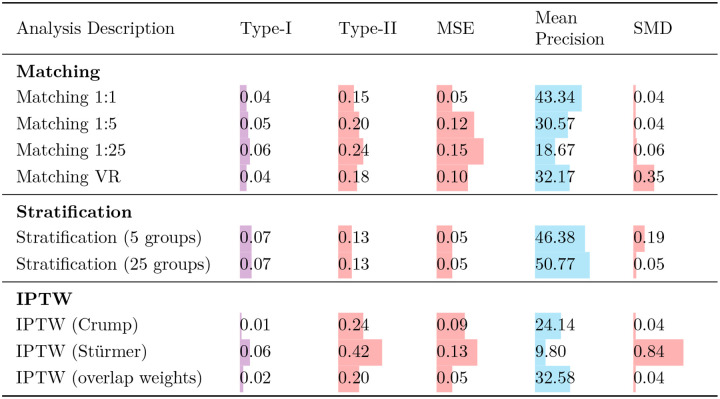
Evaluation metrics of all PS models post-calibration for new users of Metoprolol vs. Lisinopril in the Merative Marketscan Medicaide Supplemental Database (MDCD). Columns plotted in red correspond to metrics for which lower values indicate better performance, while columns in blue correspond to metrics for which higher values indicate better performance.

The case-study metrics (Figure 5) reinforce this: calibrated IPTW shows higher type-II error and lower precision than 1:1 matching, while stratification retains relatively high precision post-calibration.

## 5 Discussion

Our findings offer practical guidelines for researchers implementing LSPS models, based on empirical assessment of bias and error rates using negative and positive control outcomes across a wide range of target-comparator-outcome sets.

A key motivation for this work stems from prior literature that suggested inverse probability of treatment weighting (IPTW) may perform poorly in large-scale PS contexts [22]. In this study, we detail three specific variants of IPTW: Crump trimming, Stürmer trimming, and overlap weighting. We demonstrate that two of the three variants, Crump trimming and overlap weighting, perform very well, often surpassing matching-based strategies in terms of balancing treatment groups and controlling bias. This breakdown of IPTW methods highlights that IPTW can be a highly effective approach in large-scale observational studies, particularly when extreme weights are properly addressed.

Despite our findings on the effectiveness of IPTW, no single adjustment strategy universally outperforms the others. While IPTW (Crump) and overlap weighting consistently rank among the top performers, matching and stratification may be preferable in certain contexts, such as when higher precision is needed. In some comparisons, 1:1 matching is easily comparable to IPTW methods in terms of balance and bias control, while also showing higher precision and lower MSE. In our large-scale databases, larger matching ratios (1:25, 1:5, VR) occasionally outperformed both IPTW and 1:1 matching, suggesting the optimal ratio depends on database size and cohort composition.

Additionally, researchers should consider the targeted estimand for different adjustment strategies. Matching targets the ATT, stratification targets the ATE, and IPTW will target either the ATO (overlap weighting) or a modified ATE (trimming). Finally, we consider the idea that trimming and matching can result in the exclusion of patients, whereas stratification typically retains all patients but may be less effective at controlling bias and balancing treatment groups. Because of these factors, we hesitate to make a one-size-fits-all recommendation to researchers conducting large-scale observational studies. Based on our aggregate results, IPTW with Crump trimming can be a strong balance between bias control and variance for researchers opting to use IPTW, while 1:1 matching can be a strong default choice for those seeking a simple and robust method, often with higher precision.

One notable finding from this study was the consistent underperformance of the Stürmer trimming method across all evaluation metrics. We attribute this to the fact that Stürmer trimming removes the smallest PS estimates in the target group and the largest PS estimates in the comparator group [19]. This procedure removes a substantial portion of the overlapping region of the PS distribution between treatment groups, which is the region that other methods like Crump trimming and overlap weighting try to emphasize. Because our causal comparisons rely heavily on patients with similar treatment probabilities in both cohorts, trimming away these overlapping regions reduces comparability and can lead to biased treatment effect estimates. This directly corresponds with existing benchmark results found by Schuemie et al. [22], who also found that IPTW (Stürmer) underperformed relative to matching and stratification.

An interesting result from our analyses was that 1:1 matching sometimes yielded slightly higher precision compared to fixed 1:many or VR matching, even though both approaches achieved similar balance and MSE. In theory, increasing the number of matches should reduce variance, as additional matches increase the sample size per matched group. One explanation is that extra matches in 1:many matching may add less-similar patients, introducing variance. Additionally, 1:many and VR approaches require conditional outcome models, which account for within-set correlation at a cost to statistical power. When the comparator cohort is large and well-balanced, VR matching or stratification can offset this by leveraging additional controls. Otherwise, 1:1 matching or IPTW is preferable.

Finally, this study demonstrates that calibrating for a nominal type-I error substantially reduces differences between PS adjustment strategies across all performance metrics. We see that calibration aligns type-I and type-II error rates, precision, and balance for all strategies, excluding IPTW (Stürmer). Because of this, we suggest that calibration is an effective way to standardize performance and reduce the estimated treatment effects’ sensitivity to the choice of PS adjustment strategy. We strongly recommend researchers to consider incorporating empirical calibration [31] when applying large-scale PS models.

A common concern raised on the systematic approach for selecting many covariates in LSPS models is the potential inclusion of instrumental variables (IVs). In this study, we screened for “obvious” IVs by computing a numerical univariate correlation for each covariate on the treatment, and removing those with no causal relationship to the outcome. However, we note that in a study by Tian et al. [33], negative control experiments similar to those conducted in this study found that IV inclusion has only a weak effect on bias and precision. These findings suggest that the presence of a small number of IVs is unlikely to significantly impact the validity of treatment effect estimates in LSPS model settings.

Future directions naturally extend to doubly robust estimation strategies, commonly used with PS weighting schemes. While these approaches are theoretically appealing due to their consistency under correct specification of either the PS or the outcome model, their practical performance in large-scale settings such as those of this study are unclear. Additionally, although methodological work often emphasizes precise estimand definitions (e.g., ATE vs ATT), the practical importance of these distinctions may be limited in settings with existing substantial overlap between treatment groups. In these cases, different PS adjustment schemes target populations that are largely similar in composition, which can lead to comparable effect estimates, despite differences in the formal estimand definition. It may be valuable to quantify the point at which differences between estimands are negligible, and whether simpler weighting approaches remain sufficient for large-scale studies that can impact real-world healthcare decisions.

Overall, IPTW with Crump trimming and 1:1 matching are competitive defaults, but no single strategy dominates across all settings. We recommend evaluating top-performing strategies as sensitivity analyses alongside covariate balance diagnostics, so that researchers can assess the stability of their findings sensitivity to the PS modeling choices. Finally, we strongly recommend empirical calibration in future studies to improve reliability against poor PS model choices.

## 6 Conclusion

This study provides a set of real-world benchmarks for large-scale propensity score models, highlighting their performance across multiple observational health databases. Our results suggest that IPTW with Crump trimming and 1:1 matching can be strong default choices, showing a good balance between bias control and precision. However, we also maintain that larger matching ratios or IPTW with overlap weighting may be advantageous in specific study contexts. Additionally, we found that empirical calibration effectively converges performance across adjustment strategies, which can reduce sensitivity to the choice of PS model. Overall, our results offer data-driven guidance for researchers, and we suggest that future observational health studies carefully select the PS adjustment strategy suitable for their study goals.

## Supplementary Material

Supplementary Files

This is a list of supplementary files associated with this preprint. Click to download.

- suppmaterialslarge.pdf

## Figures and Tables

**Table 1:** Committed data sources and their covered populations

Data source	Population	Patients	History	Data capture process and short description
**Administrative claims**				
IBM MarketScan Commercial Claims and Encounters (CCAE)	Commercially insured, < 65 years	142M	2000 –	Adjudicated health insurance claims (e.g. inpatient, outpatient, and outpatient pharmacy) from large employers and health plans who provide private healthcare coverage to employees, their spouses and dependents.
IBM MarketScan Medicare Supplemental Database (MDCR)	Commercially insured, 65+ years	10M	2000 –	Adjudicated health insurance claims of retirees with primary or Medicare supplemental coverage through privately insured fee-for-service, point-of-service or capitated health plans.
IBM MarketScan Multi-State Medicaid Database (MDCD)	Medicaid enrollees, racially diverse	26M	2006 –	Adjudicated health insurance claims for Medicaid enrollees from multiple states and includes hospital discharge diagnoses, outpatient diagnoses and procedures, and outpatient pharmacy claims.
**Electronic health records (EHRs)**				
Optum^®^ de-identified Electronic Health Record Dataset (Optum^®^ EHR)	US, general	93M	2006 –	Clinical information, prescriptions, lab results, vital signs, body measurements, diagnoses and procedures derived from clinical notes using natural language processing.

**Table 2:** Number of unique patients meeting eligibility criteria in each treatment cohort across databases.

Database	Merative MDCR	Merative MDCD	Merative CCAE	Optum^®^ EHR
**Classes**				
ACE inhibitors	142,209	160,977	1,166,234	1,335,950
Angiotensin receptor blockers	57,851	40,669	485,372	569,384
Beta blockers - cardioselective	95,474	72,095	435,371	665,242
Thiazide or thiazide-like diuretics	50,142	79,904	449,462	422,300
**Drugs**				
Hydrochlorothiazide	46,587	73,321	418,810	380,513
Metoprolol	74,032	59,849	318,230	531,928
Lisinopril	113,016	153,392	1,025,539	1,222,849
Losartan	33,941	33,521	293,831	425,657
**Total**	613,252	673,728	4,592,849	5,553,823

**Table 3:** Analysis variants for large-scale propensity score (PS) models and their conditioning.

PS adjustment variant	Conditional
**Matching**	
Matching 1:1	N
Matching 1:5	Y
Matching 1:25	Y
Matching VR	Y
**Stratification**	
Stratification 5 groups	Y
Stratification 25 groups	Y
**IPTW**	
IPTW (Crump)	N
IPTW (Sturmer)	N
IPTW (overlap weights)	N

**Table 4:** Covariate balance for baseline characteristics measured by standardized mean difference (age group, gender) and imbalanced traits pre-adjustment, and post-adjustment after IPTW (Stürmer) or 1:1 matching. Results are shown for new users of Metoprolol vs. Lisinopril in the Merative MarketScan Medicaide Database (MDCD).

	Before			IPTW (Sturmer)			Matching 1:1		
Characteristic	Metoprolol (%)	Lisinopril (%)	Std. diff	Metoprolol (%)	Lisinopril (%)	Std. diff	Metoprolol (%)	Lisinopril (%)	Std. diff
**Age group**									
< 30	11.3	15.2	−0.03	10.4	9.6	−0.02	10.6	11.0	−0.01
30 – 44	26.5	30.9	−0.03	25.7	23.9	0.04	27.7	26.9	0.01
45 – 54	21.8	23.7	−0.05	21.7	19.9	0.03	22.2	21.7	0.01
55 – 64	23.7	21.8	0.01	23.9	25.7	−0.04	22.9	23.6	−0.01
> 65	16.8	8.5	0.09	18.2	20.9	−0.07	16.6	16.7	−0.01
Female	60.2	53.5	0.13	56.7	54.7	0.04	56.5	56.5	0.00
Anemia	22.1	11.4	0.29	18.4	18.9	−0.02	14.2	14.3	0.00
Angina pectoris	7.9	1.4	0.31	7.2	11.4	−0.14	3.8	3.6	0.01
Arteriosclerotic vascular disease	22.2	6.6	0.46	21.2	28.7	−0.17	14.3	14.3	0.00
Cardiac arrhythmia	25.1	6.6	0.53	22.5	28.9	−0.15	13.0	13.3	−0.01
Chest injury	21.9	7.4	0.42	19.6	25.8	−0.15	12.5	12.6	0.00
**Gender**									
Ischemic heart disease	16.9	3.6	0.45	15.7	21.9	−0.16	9.0	8.8	0.01
Myocardial infarction	10.8	2.1	0.36	10.2	15.0	−0.14	5.5	5.3	0.01
**Medical History**									
Antithrombotic agents	26.7	11.2	0.40	24.7	29.5	−0.11	17.0	16.5	0.01
Aspirin	9.3	4.7	0.18	8.5	10.0	−0.05	6.0	5.9	0.01
Psycholeptics	45.0	35.3	0.20	38.7	40.3	−0.03	36.8	38.1	−0.03
Organic nitrates	7.0	1.2	0.29	6.6	10.0	−0.13	3.4	3.3	0.00

## Data Availability

The data underlying this article cannot be shared publicly due to the privacy of individuals whose data was used in the study. Aggregated data to support the findings in this study can be made available upon a reasonable request to the corresponding author.
